# A High Salt Diet Modulates the Gut Microbiota and Short Chain Fatty Acids Production in a Salt-Sensitive Hypertension Rat Model

**DOI:** 10.3390/nu10091154

**Published:** 2018-08-23

**Authors:** Ariel Bier, Tzipi Braun, Rawan Khasbab, Ayelet Di Segni, Ehud Grossman, Yael Haberman, Avshalom Leibowitz

**Affiliations:** 1 Internal Medicine D and Hypertension Unit, The Chaim Sheba Medical Center, Tel-Hashomer, Ramat Gan 5265601, Israel; arielbier@gmail.com (A.B.); rawankhasbab@mail.tau.ac.il (R.K.); Ehud.Grossman@sheba.health.gov.il (E.G.); 2 Sackler Faculty of Medicine, Tel-Aviv University, Tel Aviv 69978, Israel; 3 The Chaim Sheba Medical Center, Tel-Hashomer, Ramat-Gan 5265601, Israel; zipik0@gmail.com (T.B.); ayeletdisegni@gmail.com (A.D.S.); Yael.Haberman@sheba.health.gov.il (Y.H.); 4 Cincinnati Children’s Hospital Medical Center, University of Cincinnati College of Medicine, Cincinnati, OH 45229, USA

**Keywords:** salt, blood pressure, microbiome, short chain fatty acids

## Abstract

Emerging data indicate a correlation between gut microbial composition and cardiovascular disease including hypertension. The host’s diet greatly affects microbial composition and metabolite production. Short chain fatty acids (SCFAs) are products of microbial fermentation, which can be utilized by the host. It has been suggested that SCFAs play a pivotal role as mediators in a microbiome host: microbial interactions occur in health and disease. The aim of this study was to evaluate the effect of a high salt diet (HSD) on microbial variation and to determine whether this effect is accompanied by an alteration in fecal SCFAs. To this end, Dahl salt-sensitive rats were divided into two groups (*n* = 10 each): (A) Control: fed regular chow; and (B) Fed HSD. High-throughput pyrosequencing of the 16S rRNA amplicon sequencing was used for microbiome characterizing. Chromatography-mass spectrometry was used to measure the levels of SCFAs: acetic acid, propionic acid, butyric acid, and isobutyric acid in fecal samples. Differences in microbial composition were noted between groups. Principal Coordinate Analysis (PCoA) principal coordinate 1 (PC1) primarily separated controls from the HSD. Four taxa displayed significant differences between HSD and controls. Taxa from the Erwinia genus, the Christensenellaceae and Corynebacteriaceae families, displayed an increased abundance in HSD versus control. In contrast, taxa from the Anaerostipes genus displayed a decreased abundance in HSD. We were able to identify seven unique taxa that were significantly associated with blood pressure. There was a significant difference in fecal acetic acid, as well as propionic and isobutyric acid, but not in the butyric acid composition between groups. Adding salt to a diet impacts the gut’s microbial composition, which may alter fecal SCFA production.

## 1. Introduction

Hypertension is a major cardiovascular risk factor that afflicts more than 1 billion people worldwide. In most contemporary societies, blood pressure (BP) levels rise steadily and continuously with age in both men and women [1]. One explanation for the increasing prevalence of hypertension in the modern world is the steady rise in salt consumption. This theory is based on many studies that showed that a high salt diet (HSD) is a major contributor to hypertension development and to kidney injury in the western population [2,3]. Although the link to hypertension and salt consumption is common knowledge, the mechanism underlying it is not fully understood and needs to be elucidated.

In diseases that are diet dependent, the interaction between gut microbiota and host metabolism plays a significant role; hence, microbiome alteration is a crucial factor [4,5]. Since diet influences hypertension, recent works revealed the link to microbiota composition and hypertension development by studying animal models of hypertension [6]. The effect of HSD on gut microbial composition has been reported in the last two years using several mouse models of various diseases [7,8,9,10]. Hu et al. [7] have studied the mechanism underlying high salt diet-induced renal injury and hypertension. They described changes in the intestinal microflora that induced intestinal immunological gene expression and gut permeability. Furthermore, they detected enteric bacterial translocation into the kidney. Wilck et al. [10] have studied the microbiota of HSD-induced hypertension and have shown that a specific bacterium, *lactobacillus murinus,* is affected. They have also demonstrated that treatment using this bacterium prevents salt-induced hypertension. One of the common models of HSD-induced hypertension is the Dahl salt-sensitive (DSS) rat model. In this model, BP elevation and kidney injury by HSD is more prominent than in the mouse model, which has been used in previous studies [11,12]. Mell et al. [13] were the first to study the gut microbial composition of Dahl rats. They have shown differences in the gut bacterial composition between salt-sensitive and salt-resistant Dahl rats. Interestingly, they have shown that fecal transplantation from one strain to another may have changed the BP pattern. However, their study focused on the baseline microbial composition of a normal diet and not on the effect of HSD.

Short chain fatty acids (SCFAs), which are products of the fermentative activity of gut bacteria, are suggested as a mediator of the interaction between gut microbiota and host [14]. Pluznick [15] demonstrated in several studies that novel receptors of SCFA are involved in BP regulation, supporting the possibility that SCFAs are important mediators between microbiota and host BP regulation.

In a recent review, a working group (WG) from the National Heart, Lung, and Blood Institute identified several major scientific issues that should be addressed in the near future. One of these issues is “Kidney–gut axis in hypertension, mechanisms, gut pathophysiology, and implications in development of hypertension” [16].

Taken together, to the best of our knowledge, no data exist regarding the effect of HSD on gut microbial composition in the classic salt-induced hypertension model. Some intriguing open questions are as follows: Does HSD affect DSS gut microbial composition? Does it affect SCFA production?

Thus, the aim of this study was to evaluate how a high salt diet alters the gut microbiota and SCFA production in comparison to a regular diet.

## 2. Materials and Methods 

### 2.1. Animals and Study Protocol

The study protocol was approved by the institutional animal ethics committee (941/14). Male Dahl salt-sensitive rats, 4 weeks old (Harlan, Indianapolis, IN, USA), were housed in regular cages (2 rats per cage) in an animal room at 22 °C with a 14-h light/10-h dark cycle. Rats were maintained on a normal salt diet and were given tap water to drink ad libitum for an acclimation period of 5 days. The rats were then divided into two groups (*n* = 8–10 each), according to diet, over an 8-week period. The control group was fed a normal salt diet and tap water and the HSD group was fed an enriched salt diet and tap water. Tail cuff BP (using an electrosphygmomanometer and a pneumatic pulse transducer (58500 BP Recorder; UGO BASILE, Varese, Italy)) were measured every 2 weeks throughout the treatment. During the last week of treatment, over a 24-h period, rats were weighed and then were placed in metabolic cages for collection of urinary protein and feces.

### 2.2. Diets

The normal salt diet (0.5% NaCl diet (2018SC; Teklad Envigo Madison, WI, USA)) consisted of 44.2% carbohydrates, 18.6% protein, 6.2% fat, and a standard vitamin and mineral mix. The energy density was 3.1 Kcal/g. The HSD (4% NaCl diet (TD92034; Teklad Envigo, Madison, WI, USA)) consisted of 46.7% carbohydrates, 18.5% protein, 5.3% fat, and a standard vitamin and mineral mix. The energy density was 3.1 Kcal/g. The diets were comparable in terms of calorie and macronutrient composition. A detailed comparison of the diets is provided as supplemental data (SUPP 1).

### 2.3. DNA Extraction, PCR Amplification and Sequencing

Fresh fecal samples were collected during the last week of the experiment for gut microbial characterization. Bacterial genomic DNA was extracted from frozen fecal samples stored at −80 °C. A smear approximately 5 mm^2^ (roughly the size of a pencil eraser) was taken from a fecal sample using a sterile swab in a test tube. DNA extraction and PCR amplification of variable region 4 (V4) of the 16S rRNA gene using Illumina-adapted universal primers 515F/806R was conducted using the direct PCR protocol (Extract-N-Amp Plant PCR kit (Sigma-Aldrich, Inc., St. Louis, MO, USA)) as previously described [17,18]. Briefly, PCRs were conducted in triplicate in 96-well plates (denaturation for 3 min at 94 °C; 35 cycles (98 °C, 60 s; 55 °C, 60 s; 72 °C, 60 s) followed by elongation for 10 min at 72 °C). Positive amplicons were pooled in equimolar concentrations into a composite sample that was size selected (300–500 bp) using agarose gel to reduce non-specific products from the host DNA. Sequencing was performed on the Illumina MiSeq platform (San Diego, CA, USA) with the addition of 20% PhiX (illumina cat #15017666, San Diego, CA, USA) and paired-end reads of 175 b in length were generated in each direction.

### 2.4. Data Processing and Analyses 

The sequenced fastq files were processed in a data curation pipeline implemented in QIIME 1.9.1 (QIIME enables the analysis of high-throughput community sequencing data) [19]. Paired-end reads stitched together and reads with an overlap of fewer than 50 nucleotides or more than a 5% difference within the overlap region were excluded. Further quality control was performed by truncating the joined reads at nucleotides with a Phred score lower than 30. Reads with ambiguous characteristics or less than 75% of the original length after truncation were discarded. The remaining reads were binned by a sample-specific barcode, with no barcode errors. Sequences that passed quality filtering were clustered into phylotypes (Operational Taxonomic Units, OTUs) at 97% sequence identity using a uclust-based [20] closed-reference protocol, against the Greengenes database [21] (August 2013 version). Reads that did not match a sequence in the reference set with at least 97% identity were excluded from subsequent analyses. The taxonomy of each phylotype was assigned as the taxonomy associated with the Greengenes sequence defining that OTU. The Greengenes phylogenetic tree was used for phylogenetic diversity calculations. A median of 10,932 (an average of 11,153) paired-end sequences were collected per sample. All samples were randomly rarefied to 2000 sequences for downstream β diversity analysis. Weighted and unweighted UniFrac distance was used as a measure of β-diversity or between-sample diversity. The resulting β-diversity distance matrix was used to perform Principal Coordinates Analysis (PCoA) analysis. Taxonomic analysis was performed on the relative abundance table, to avoid the effect of sample size. QIIME scripts/summarize_taxa.py was used to calculate the relative abundance table. Downstream data analyses were performed in R. Univariate analyses were performed using LEfSe (Linear discriminant analysis (LDA) Effect Size,) Galaxy version 1.0 with default parameters. They were used to check for an association between fecal microbial compositions in rats receiving salt in comparison to control rats. Multivariate Association with Linear Models (Maaslin) R package version 0.0.5 was used [22,23] to find taxa significantly associated with blood pressure measurements. Taxa up to the genus level were tested, and any taxa that did not have at least one read in 10% of the analyzed samples were removed. Only significant associations with *q* ≤ 0.1 after false discovery rate (FDR) correction were included.

### 2.5. Short Chain Fatty Acids Extraction and Analysis

Flash-frozen fecal contents (100 mg) were mashed in 2 mL acidic water (PH = 2.4) and centrifuged at 12,100× *g* for 20 min at 4 °C, after which the supernatants were taken for analysis. The samples were mixed with 10 µmoL/g internal standards (acetic-d4 acid, Sigma-Aldrich #151785; propionic-3,3,3-d3 acid, #486159; and butyric-d8 acid, #588555). Two rounds of extraction using 1 mL hexane were carried out by mixing for 10 min at room temperature, followed by centrifugation at 1932× *g* for 10 min at 4 °C. Extracts were then incubated at 60–70 °C for 45 min with *N*-*tert*-Butyldimethylsilyl-*N*-methyltrifluoroacetamide (MTBSTFA) (Sigma-Aldrich (6 microliter in a 180 microliter sample. Gaschomtography−mass spectrometry (GC−MS) analyses were performed on an Agilent 6890/5977A GC-MS (Santa Clara, CA, USA) system equipped with Agilent 30 m × 0.25 mm i.d. HP-5MS UI column (5% Phenyl/Methylpolysiloxane, 0.25 μm film thickness). The carrier gas was helium (99.999%) at a constant flow rate of 1.0 mL/min. The GC conditions were as follows: injection volume 1.0 μL (Agilent auto-sampler G4513A, China); injector temperature 250 °C in splitless mode; the initial oven temperature was 60 °C, which was maintained for 4 min, and increased to 80 °C at a rate of 2 °C/min, which was followed by raising the temperature to 260 °C at a rate of 30 °C/min for 3 min. MS was performed in the EI positive ion mode at 70 eV electron energy. Transfer line temperature and ion source temperature were maintained at 280 °C and 250 °C, respectively. MS data were collected in full-scan mode (*m*/*z* 60−300) and analyzed with Agilent Chemstation software (Agilent Technologies, Ver. F.01.01.2317). The m/z of reconstructed single ion monitoring (RSIM) are as follows: 117 (acetic acid), 120 (acetic-d4 acid), 131 (propionic acid), 134 (propionic-3,3,3-d3 acid), 145 (butyric acid), and 152 (butyric-d7 acid).

## 3. Results

### 3.1. Blood Pressure and Proteinuria

**Figure 1:**
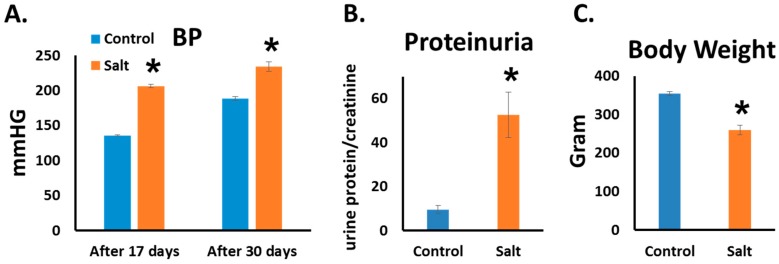
High salt diet (HSD)-fed rats developed hypertension accompanied by kidney injury. Blood pressure (BP) was measured using tail cuff. (The results of the 2nd and 5th weeks are shown.) (**A**) Proteinuria was measured in rat’s urine at the end of the 8th week; (**B**) Body weight was measured at the end of the experiment (the 8th week); (**C**) *n* = 8–10, * *p* ≤ 0.05.

As is already known from previous works from our labs and others, HSD induces hypertension and kidney injury in DSS rats [11,12,24]. In this study we observed the same effect of HSD. BP levels were significantly higher in HSD than in controls. Significant differences were already noted after two weeks (206 ± 3 vs. 136 ± 1 mmHg, HSD vs. control) and were maintained throughout the study (234 ± 7 vs.185 ± 4 mmHg, HSD vs. control, on 5th week) (Figure 1A). By the end of the study, urinary protein excretion was significantly higher in HSD than in the control (52.5 ± 10 vs. 9 ± 1.8 24 h urine protein/creatinine, respectively) (Figure 1B). Furthermore, HSD-fed rats exhibited a significant weight reduction in comparison with rats fed the control diet (Figure 1C)**.**

### 3.2. The Microbiome Profile

**Figure 2:**
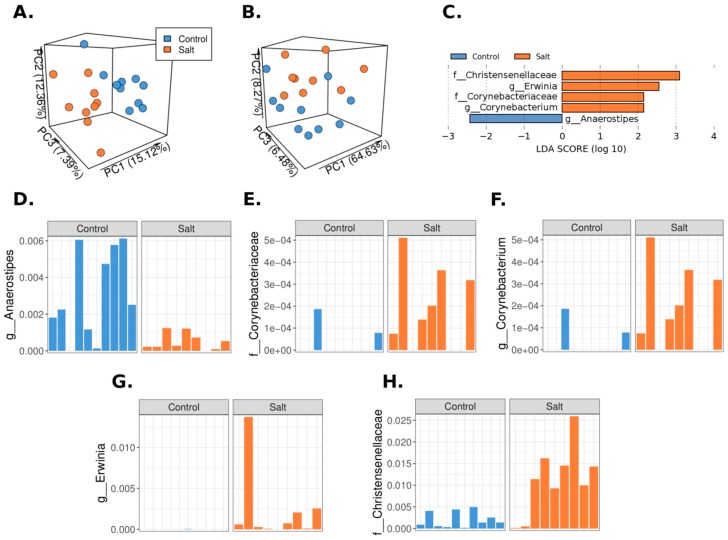
Microbial composition in high salt diet (HSD) differs from the control. Fecal pellets from HSD and control rats were analyzed using 16S rRNA-Amplicon Sequencing. Unweighted (**A**) and weighted (**B**) UniFrac-based Principal Coordinates Analysis (PCoA) analysis of the cohort was performed to visually explore the similarity and variations between the samples’ microbial composition. PCoA indicated that a visual separation exists between HSD and control samples; (**C**) Five different microbial taxa displayed significant abundance differences (Linear discriminant analysis (LDA) score > 2) between HSD and control, using the LDA Effect Size analytic approach; (**D**–**H**) The relative abundance of the 5 significant microbial taxa in the LDA Effect Size analysis is shown for each sample.

To determine the effect of salt on the microbiome composition, we analyzed fecal pellets from HSD-fed rats and controls by 16S rRNA-Amplicon Sequencing. Briefly, an unweighted and weighted UniFrac-based PCoA of the cohort was performed to visually explore the similarity and variation between the samples’ microbial composition (Figure 2A,B). The HSD cases are predominantly clustered on one side of the plot, and the controls are on the other side, where most of the separation was driven by the PC1 values in the unweighted and UniFrac-based PCoA and by PC2 in the weighted PCoA plot. Univariate analysis (LDA Effect Size) was performed to check for an association between the microbial composition and HSD-fed rats and controls. Five taxa exhibited significant differences between HSD and control (Figure 2C). Taxa from the *Erwinia* genus, the *Christensenellaceae,* and *Corynebacteriaceae* families displayed an increased abundance in HSD versus control. In contrast, taxa from the *Anaerostipes* genus displayed a decreased abundance in HSD-fed rats (Figure 2D–H).

**Figure 3:**
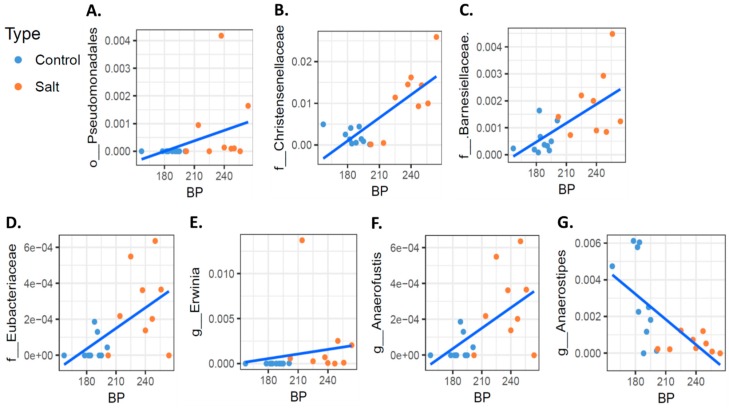
Correlation between blood pressure (BP) and bacterial taxa. Rat’s BP values after 30 days were analyzed for their correlation with specific bacterial taxa; 7 microbial taxa displayed a significant correlation (*q* value ≤ 0.1) with BP (**A**–**G**) using the Multivariate Association with Linear Models (Maaslin) package. Blue dots and red dots depict fecal samples from the high salt diet and controls, respectively.

Interestingly, using a Multivariate Association with Linear Models (MaAsLin) pipeline [19], which can handle continuous values to identify microbial taxa that were significantly associated with BP, we were able to identify seven unique taxa. These included taxa of the *Pseudomonadales* order, the *Christensenellaceae*, *Barnesiellaceae,* and *Eubacteriaceae* families, and of the *Erwinia* and *Anaerofustis* genus (Figure 3A–F). In contrast, we identified a significant negative correlation between BP and taxa of the *Anaerostipes* genera (Figure 3G).

### 3.3. Short Chain Fatty Acid

**Figure 4:**
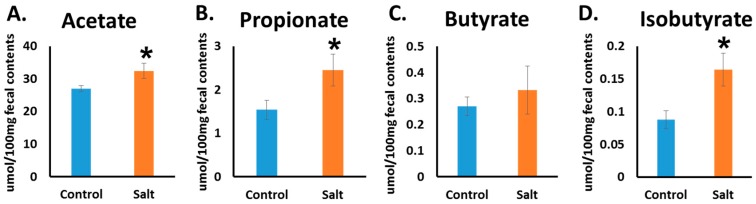
High salt diet elevated the SCFA level. The level of acetate (**A**); propionate (**B**); butyrate (**C**), and isobutyrate (**D**) were measured by GC-MS from fecal pellets at the end of the experiment. *n* = 8–10, * *p* ≤ 0.05.

One of the mechanisms by which microbiota affect human health and disease is its capacity to produce SCFAs by fermenting dietary fibers. The SCFAs are likely to have broad impacts on various aspects of host physiology. Hence, this led us to investigate the changes in the SCFA level in the rat’s fecal pellets. Using GC−MS, we measured the levels of SCFAs: acetate, propionate, isobutyrate, and butyrate. HSD significantly elevates acetate, propionate, and is obutyrate levels in comparison with the control. For butyrate, no significant changes were observed (Figure 4A–D). We looked for a correlation between bacterial taxa and SCFA changes; we observed a negative correlation between the taxa of the phylum *Actinobacteria* and the butyric acid level, independently of the diet change (data not shown).

## 4. Discussion

Essential hypertension is strongly correlated with kidney disease, and salt consumption has consistently and repeatedly been implicated in the development of these conditions. The Western diet contains a high salt content, which induces hypertension and kidney damage [2]. In our study, we used the DSS rat model, which develops kidney injury and hypertension, as a result of consuming a high salt diet. We investigated whether a high salt diet affects the gut microbial composition.

Importantly, we were able to show that HSD alters the gut microbiome composition. Unweighted and weighted UniFrac-based Principal Coordinates Analysis (PCoA) revealed an overall separation between HSD (*n* = 9) and control (*n* = 10) groups. However, at the taxa level, we found that only few taxa’s relative abundance were appreciably different. These findings may indicate a high microbial variation between cases, with significant changes attributed to only a few specific taxa. Importantly, similar to our animal model, a previous study already linked chronic kidney disease (CKD) to *Corynebacterium*/*Corynebacteriaceae* taxa, which were upregulated in chronic kidney disease [25].

We identified a significant correlation between BP values and seven microbial taxa at the genus and family levels. The presence of such a correlation is novel and this was not studied extensively. We found only a few very recent studies that have shown this kind of correlation. Hidalgo et al. described the microbiome changes that a diet enriched with olive oil induced in the spontaneous hypertensive rat model. Among other findings, they found a negative correlation between systolic blood pressure and the relative abundance of clostridia XIV [26]. However, in this study they looked for only a few bacteria. In a study that investigated the human microbiome difference between hypertensive patients versus matched controls, Kim et al. found several bacterial taxa that positively and negatively correlate with BP [27]. Nevertheless, to the best of our knowledge, we are the first to report a strong correlation between BP and specific bacteria’s relative abundance in salt-induced hypertension. The effect of HSD on microbiome and hypertension has been recently studied by Wilck and colleagues [10] in FVB/N mice. Mice were fed a normal salt diet or HSD for 14 days. They identified several taxa that were significantly decreased or increased after a HSD diet. Interestingly, there is no overlap with our findings for these bacterial taxa. The differences between the studies may result from using different animal models, different amounts of salt in the diet, and a shorter period of feeding salt. By using a sensitive machine learning approach to distinguish between control and HSD, they identified eight taxa. One of the taxa is of the class *Clostridia,* which is down-regulated in HSD. In our study, we did not find a significant change in the entire class; however, two out of four of the taxa in which the control was distinguished from HSD belong to the *Clostridia* class, including taxa of the genera *Anaerostipes,* which were down-regulated in HSD, and of the family *Christensenellaceae,* which were up-regulated in HSD. In addition, four bacterial taxa that were correlated with BP are also subgroups of the *Clostridia* class, including the family *Christensenellaceae* and *Eubacteriaceae* and the genera *Anaerofustis,* all of which correlate positively with BP, and the genera *Anaerostipes,* which correlates negatively with BP.

In addition to Wilck’s [10] study, there are a few more studies that evaluated the effect of HSD on gut microbial composition. Miranda et al. [9] studied a mouse model of HSD-induced colitis, and Wang et al. [8] studied only the microbiome changes and inflammation. However, in these studies there are no BP data. Hu et al. [7] also conducted a study that evaluated the effect of HSD on gut microbiota, gut leakage, and the effect on the kidneys. They also used a mouse model. The salt content in these studies ranged from 2 to 5% salt. We preferred to study an intensive BP model; thus, we choose the DSS model and a moderate percentage of diet salt, as we used in our previous study with this model [24]. With this model there are studies that used even 8% salt content [28].

In our study we found that HSD-fed rats, which developed hypertension, have higher levels of SCFAs (in particular, acetate, propionate, and isobutyrate, but not butyrate) in fecal samples than do controls. The relevance of SCFAs to BP regulation was demonstrated in a previous study by Pluznick et al. [15] who showed the presence of a specific SCFA receptor in kidneys: Olfactory-78. In addition, they demonstrated that ligation of SCFAs to this receptor may alter BP. According to the literature, acetate and propionate are the ligands of olfactory-78 [29]. Hence, our finding may be relevant to the development of hypertension in our model.

Mell et al. [13] conducted a complex study in order to determine the role of microbiome in the development of hypertension in DSS rats. They also measured SCFA levels and found elevated levels of acetate in DSS fed HSD, as we found in our study. However, they measured it in plasma and we measured it in the fecal samples. In addition, they did not measure the levels of proprionate and butyrate as we did in our study, but instead, measured other SCFAs [13]. Other studies reported that SCFAs may lower BP (for a review, see [29]). The role of SCFA levels in hypertension is still not clear and more studies should be conducted to clarify it. In our study, there was no significant correlation between any of the SCFAs that were altered in HSD and specific bacterial taxa. Consequently, we speculate that no specific bacterium induced the SCFA changes, but instead, all of the changes in composition had contributed to inducing these changes.

The bacteria of the genera *Anaerostipes* have shown a negative correlation with BP; this bacterium is described in the literature as an acetate-utilizing, butyrate-producing bacterium [30], which is in agreement with our findings regarding the high levels of acetate in HSD. However, we did not find a significant correlation for acetate or any other SCFA levels with the taxa of the genera *Anaerostipes*.

Our study has several limitations. It is possible that to account for the microbial variation, large groups are required to capture more differences between tested groups. Additionally, we found a correlation between seven taxa and BP values but we do not reveal the mechanism by which those specific taxa are related to BP. We also suggest that the effect of HSD is related to SCFA production, however, we did not measure the plasma SCFA levels, which may contain more data to support it. Other authors suggested other potential mechanisms including effects on immune modulation, gut leakage, and bacterial translocation to kidneys [7] that may account for mechanistic correlation between salt, hypertension, and microbial variations.

## 5. Conclusions

In conclusion, we demonstrated in this study a significant association between HSD and gut microbial composition in the DSS rat model. We identified four taxa that are different between the normal salt and high salt diet (the taxa of the *Christensenellaceae* and the *Corynebacteriaceae* families and the *Erwinia* and the *Anaerostipes* genus). In addition, we demonstrated that in this model there was a correlation between the relative abundance of seven taxa and the BP values (including the taxa of the *Pseudomonadales* order, the *Christensenellaceae*, the *Barnesiellaceae,* and the *Eubacteriaceae* families, and of the *Erwinia* and the *Anaerofustis* genus). These observations support the notion that an interaction exists between diet salt content, gut microbial composition, and BP. Moreover, the observation that HSD is also related to changes in the fecal levels of SCFAs may highlight another aspect of the complex diet-gut-BP interaction.

## Supplementary Materials

The following are available online at http://www.mdpi.com/2072-6643/10/9/1154/s1.
