# Supplementary material for: A High Salt Diet Modulates the Gut Microbiota and Short Chain Fatty Acids Production in a Salt-Sensitive Hypertension Rat Model
Source: Nutrients. 2018 Aug 23;10(9):1154. doi: 10.3390/nu10091154 (PMC6164908; doi:10.3390/nu10091154)
Supplement: Supplementary file 1 [file nutrients-10-01154-s001.pdf]

## Suppl. 1- Control diet (2018S) and high salt diet (TD.92034) comparison

### Macronutrients.

| Diet | Control (2018s) | HSD (TD.92034) |
|---|---|---|
| Protein % | 18.6 | 18.5 |
| Fat % | 6.2 | 5.3 |
| Carbohydrate (available) % | 44.2 | 46.7 |
| Fiber (NDF) % | 14.7 | 11 |
| Energy Density (kcal/g) | 3.1 | 3.1 |
| NaCl % | 0.5 | 4.0 |

### Protein sources.

| Diet | Control (2018s) | HSD (TD.92034) |
|---|---|---|
| Primary | Soybean meal, corn gluten meal | Soybean meal, wheat |
| Secondary | Corn, wheat, wheat midds | Corn, corn gluten meal, , alflafa meal |

### Fat sources.

| Diet | Control (2018s) | HSD (TD.92034) |
|---|---|---|
| Added | Soybean Oil | Corn Oil |
| Other | Corn, wheat, wheat midds | Corn, wheat |

### Fiber sources.

| Diet | Control (2018s) | HSD (TD.92034) |
|---|---|---|
| Major | Wheat, Wheat midds | Wheat, corn |
| Intermediate | Corn | soybean meal |
| Minor | soybean meal | Alfalfa meal |

### Carbohydrate sources.

| Diet | Control (2018s) | HSD (TD.92034) |
|---|---|---|
| Major | Corn, wheat | Corn, wheat |
| Intermediate | Wheat midds | soybean meal |
| Minor | Corn gluten meal, soybean meal | Corn gluten meal, alfalfa meal |
